# Infiltrating syringomatous eccrine adenoma of the nipple: a case report

**DOI:** 10.1186/1757-1626-2-9118

**Published:** 2009-11-30

**Authors:** Deba P Sarma, Todd Stevens

**Affiliations:** 1 Department of Pathology, Creighton University Medical Center, Omaha, NE 68131, USA

## Abstract

**Background:**

Differential diagnosis for a nodule in the nipple or subareolar area of woman includes both primary neoplasms of breast as well as those from skin and adnexae.

**Case Presentation:**

A 32-year-old woman presented with a painless 0.5 cm subareolar nodule of her left nipple that she had noticed for several months, with no associated nipple discharge. A biopsy revealed an infiltrating adnexal neoplasm with features similar to those seen in syringomas commonly occurring in locations such as upper face and pubis. The infiltrating syringomatous adenoma of the nipple occurs almost exclusively in women of all ages and is cured by simple excision. Microscopic appearance of such a rare benign infiltrating neoplasm of eccrine duct origin occurring in woman's breast should not be misinterpreted as more common infiltrating primary breast carcinoma.

**Conclusion:**

Infiltrating eccrine syringomatous adenoma should be included in the differential diagnosis of a nipple or subareolar nodule occurring in woman.

## Case Presentation

A 32-year-old woman presented with a complaint of a painless 'bump' on her left nipple that she had noticed for several months. There was no history of discharge from the nipple. On physical examination, the nipple was normal. Within the areolar region, adjacent to the nipple, an ill-defined, indurated, non-tender subcutaneous 0.5 cm nodule was palpated. Clinical differential diagnoses included: leiomyoma, dermatofibroma, dermal cyst or breast tumor. A biopsy was performed.

**Figure 1:**
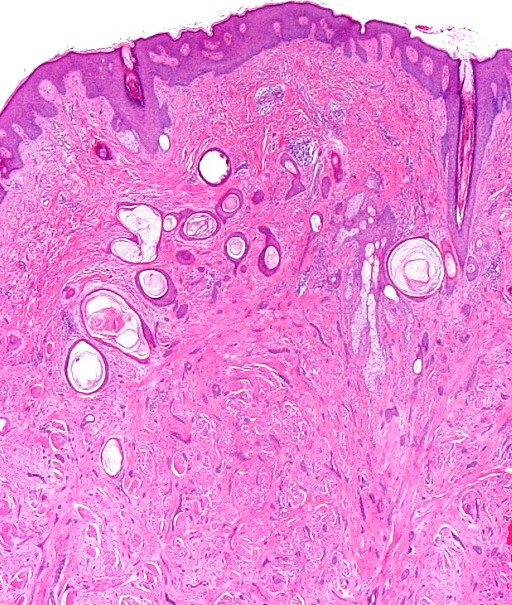
**An infiltrating dermal tumor with slightly acanthotic epidermis composed of infiltrating strands, tubules and duct-like structures within fibrotic stroma**. There are several keratotic cysts in the upper part of the tumor.

**Figure 2:**
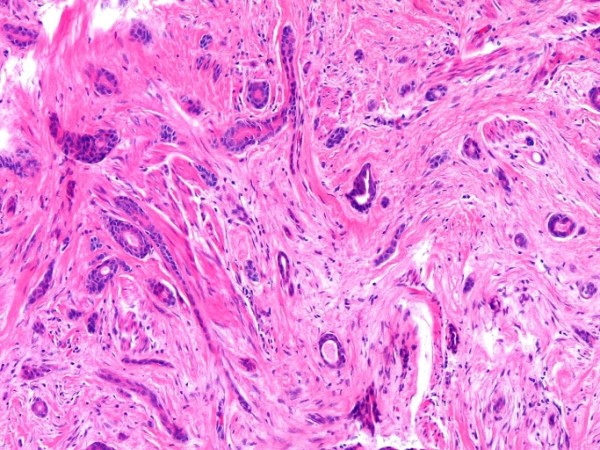
**Small, solitary, evenly spaced duct-like structures within fibrotic stroma, some demonstrating tadpole-like appearance**.

**Figure 3:**
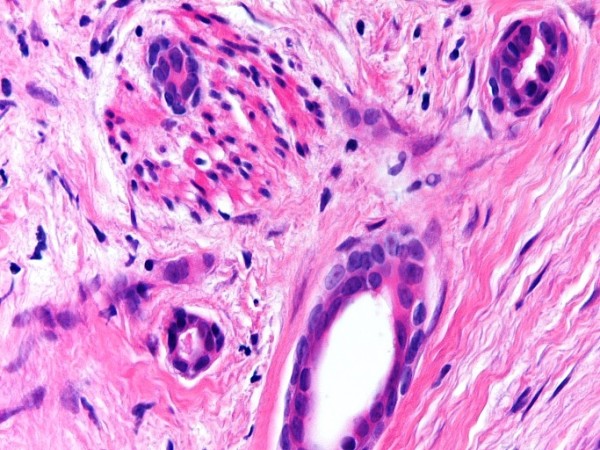
**The ducts are lined by one or more layers of cuboidal or squamoid epithelial cells**.

**Figure 4:**
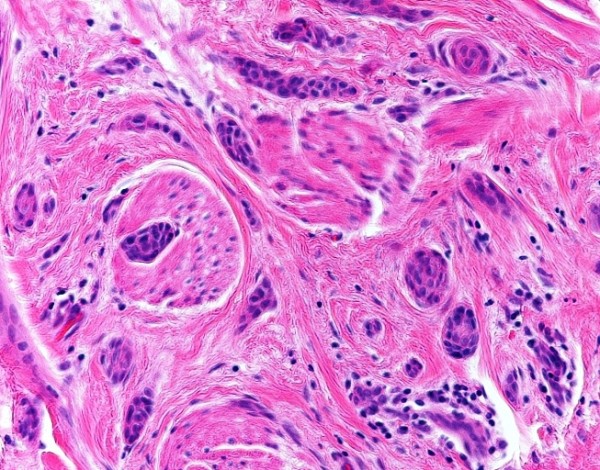
**Small, dark tumor cells are noted around and within the smooth muscle bundles of the nipple**.

**Figure 5:**
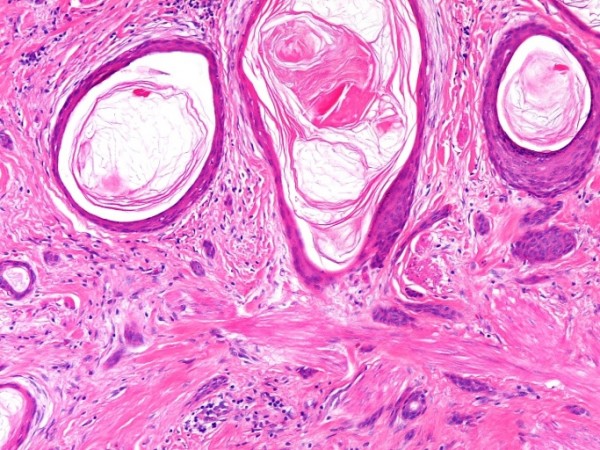
**The keratotic cysts are lined by squamous epithelium and the keratotic material within the cyst appears to be non-trichilemmal (basket-weave) type**.

Microscopically, the dermis showed an infiltrating epithelial tumor composed of small, compressed strands and duct-like structures within a fibroblastic stroma (Figure 1). The duct-like structures were solitary, well-spaced and lined by one or more layers of cuboidal or squamous cells (Figure 2). Some ductal lumina contained acellular pink material. The epithelial cells were bland and they did not display any significant pleomorphism, mitotic activity or necrosis (Figure 3). The tumor cells infiltrated around and within the smooth muscle bundles of the areola (Figure 4). The overlying epidermis showed acanthosis. In the upper part of the tumor, multiple squamous epithelial-lined cysts containing non-compact keratinous material were noted (Figure 5).

A diagnosis of infiltrating syringomatous adenoma of the nipple was made.

A complete local excision has been planned.

## Discussion

Infiltrating syringomatous adenoma of the nipple is a rare, benign, infiltrating neoplasm of eccrine origin located in the dermis of the areola and nipple region first described by Rosen in 1983 [1]. Lack of metastasis is the rule, but local recurrences can occur when the neoplasm is incompletely excised. Clinically the lesion forms a sub-areolar nodule with variable pain, erythema, nipple distortion, and nipple discharge [2]. The lesion is almost exclusively present in females, with only one reported case occurring in a male [1].

Grossly, infiltrating syringomatous adenoma of the nipple is typically a sub-areolar dermal nodule. Histologically, the epidermis may show variable acanthosis and pseudo-epitheliomatous hyperplasia. Within the dermis there is an infiltrate of curved, ductular structures lined by flattened-to-cuboidal cells, often with proteinaceous debris and skin-type keratin seen in their lumens. The lesion may involve adjacent lactiferous ducts and keratinous cysts are frequently seen. The comma-shaped ducts infiltrate amongst smooth muscle bundles and nerves, simulating a malignant mammary neoplasm. Unlike sclerosing syringomatous carcinoma (sclerosing sweat duct carcinoma), which is typified by deep, vertical stromal infiltration and prominent peri-neural spread, the infiltrating ductules of infiltrating syringomatous adenoma of the nipple show a broad-based and horizontal pattern of infiltration [3]. Histological features of tubular carcinoma of the breast including angulation of tubules and adjacent ductal carcinoma in-situ, are lacking in infiltrating syringomatous adenoma of the nipple. The hyperplasia of florid papillomatosis of the nipple and the nuclear pleomorphism, necrosis, and increased mitotic index seen in invasive ductal carcinoma are absent in infiltrating syringomatous adenoma of the nipple. The epithelial cell proliferation and the prominent stromal and basement membrane elements seen in adenoid cystic carcinoma are lacking in infiltrating syringomatous adenoma of the nipple [4]. Histologically, infiltrating syringomatous adenoma of the nipple is not much different from the syringoma that typically occurs on the lower eyelids and pubic area of women.

Complete excision is curative, with local recurrences occurring in 30% of incompletely excised lesions. A single case of possible micrometastasis to a sentinel lymph node from such a tumor has been reported [5]. Infiltrating syringomatous adenoma of the nipple clinically simulates an underlying mammary malignancy and therefore typically comes to the attention of the surgeon and surgical pathologist rather than the dermatologist and dermatopathologist. Some patients had undergone mastectomy because of misinterpretation of the lesion as a tubular carcinoma [6] or an adenosquamous carcinoma [2]. Infiltrating syringomatous adenoma of the nipple is a rare, benign, locally infiltrating neoplasm of eccrine origin that should enter into the differential diagnosis of any sub-areolar nodule.

## Competing interests

The authors declare that they have no competing interests.

## Authors' contributions

DS conceived, drafted and submitted the manuscript. TS revised the manuscript. Both authors have read and approved the final manuscript.

## Consent

Written consent was obtained from the patient for publication of this case report. A copy of the written consent is available for review by the Editor-in-Chief of this journal.
